# Experimental Study on the Mechanical Properties of Disposable Mask Waste–Reinforced Gangue Concrete

**DOI:** 10.3390/ma17040948

**Published:** 2024-02-18

**Authors:** Yu Yang, Changhao Xin, Yidan Sun, Junzhen Di, Fankang Meng, Xinhua Zhou

**Affiliations:** 1 College of Civil Engineering, Liaoning Technical University, Fuxin 123000, China; yangyu9300@163.com (Y.Y.); dijunzhen@126.com (J.D.); mfk3999147@126.com (F.M.); gpszxh@163.com (X.Z.); 2 College of Civil and Marine Engineering, Jiangsu Ocean University, Lianyungang 222000, China; 2020000083@jou.edu.cn

**Keywords:** disposable masks, gangue concrete, strength properties, energy evolution, damage constitutive

## Abstract

This paper is grounded on the following information: (1) Disposable masks primarily consist of polypropylene fiber, which exhibits excellent flexibility. (2) China has extensive coal gangue deposits that pose a significant environmental hazard. (3) Coal gangue concrete exhibits greater fragility compared to regular concrete and demonstrates reduced resistance to deformation. With the consideration of environmental conservation and resource reutilization, a preliminary concept suggests the conversion of discarded masks into fibers, which can be blended with coal gangue concrete to enhance its mechanical characteristics. In this paper, the stress–strain law of different mask fiber–doped coal gangue concrete (DMGC) under uniaxial compression is studied when the matrix strength is C20 and C30, and the effect of mask fiber content on the mechanical behavior and energy conversion relationship of coal gangue concrete is analyzed. The experimental results show that when the content of mask fiber is less than 1.5%, the strength, elastic modulus, deformation resistance, and energy dissipation of the concrete increase with mask fiber content. When the amount of mask fiber is more than 1.5%, because the tensile capacity and energy dissipation level of concrete produced by the mask fiber cannot compensate for the compression and deformation resistance of concrete of the same quantity and because excess fiber is difficult to evenly mix in the concrete, there are pore defects in concrete, which decreases the concrete strength due to the increase in mask fiber. Therefore, adding less than 1.5% mask fiber helps to improve the ductility, toughness, impermeability, and oxidation and control the cracking of coal gangue concrete. Based on Weibull theory, a constitutive model of DMGC is established, which fits well with the results of a uniaxial test, providing support for understanding the mechanical law of mask fiber–doped concrete.

## 1. Introduction

The actual demand for energy in China is increasing with continuous economic development. Although the use of new energy has become increasingly widespread, traditional coal still occupies an important position in energy consumption. In 2021, the total production of primary energy in China was 4.33 billion tons of standard coal, of which coal accounted for as much as 67.0% [1]. In 2021, China’s coal production was 4.13 billion tons, an increase of 13.2% over 2012, and the consumption was 4.23 billion tons, an increase of 20.3% over 2012 [2]. Coal is still the most important disposable energy source in China, and the healthy development of the coal industry is related to the sustainable development of China’s economy, while the eco–environmental problems caused by the development and use of coal resources have become increasingly prominent [3,4,5,6,7]. Coal gangue is a waste material that is generated during the extraction and purification of coal reserves. The production of this resource accounts for approximately 10% to 30% of the total coal output and shows a consistent annual growth of approximately 700 million tons [8].

Since the emergence of the new coronavirus (COVID–19) in 2019, until the pandemic became more common, there has been a significant rise in the utilization of healthcare protective gear, particularly single–use medical masks (DMMs). Many masks are discarded at will after use and decompose into plastic waste in rivers, oceans, and other water systems. Microplastics enter human beings and organisms with the water cycle, endangering their health and safety [9]. Some masks are burned for power generation, producing harmful gases that pollute the air and damage people’s living environment, which is not conducive to the concept of sustainable green development [10]. A mask is generally composed of three layers and is composed of polypropylene or polyester nonwovens contacting the skin layer and melt–blown ultrafine polypropylene fiber for the filter layer [11]. Because mask waste has polypropylene as the main component, it takes at least 25 years to decompose in landfills. A significant amount of mask waste causes a surge in marine pollution, threatening marine life [12,13,14,15,16] and ultimately reducing the sustainability of an ecosystem. Mask waste is typically handled in a combined manner, as opposed to being treated solely as biomedical waste. The collection and deposition of infectious waste is a major health risk in developing countries. As toxic gases (dioxins and furans) are produced during the incineration of plastics, better treatment methods should be chosen to provide full play to their value. Because the mask itself is composed of polypropylene and its mechanical properties are superior, it can be used as waste. Utilizing disposable medical masks as strengthening fibers not only enhances the structural characteristics of coal gangue concrete but also mitigates the impact of the epidemic on the living surroundings. It is estimated that nearly 2.2 billion masks are used in Asia every day, and China faces approximately 468 tons of waste related to discarded masks every day. It is proposed that the chopped mask waste should be added to recycled concrete aggregate as a filler of the pavement base or subbase to effectively improve the compressive strength, stiffness, plasticity, and flexibility of mixed fillers [17].

Coal gangue, which constitutes approximately 20% of coal production, is the waste material generated during coal mining. However, the utilization of coal gangue remains relatively low. According to incomplete statistics, the number of coal gangue hills stacked by coal gangue in China is as high as 2000, covering an area of more than 200,000 mu, and it is still growing rapidly at a rate of more than 800 million tons per year, which amounts to a great waste of resources and poses a major hidden danger to the safety and environment around the stacking area. Recently, the awareness of environmental protection and relevant laws and regulations has been deeply rooted in the hearts of the people, and the shortage of sand and gravel materials in the construction market is becoming increasingly severe. Furthermore, the utilization of coal gangue as a resource is inevitable, and the combination of the aforementioned factors establishes a prevailing inclination towards substituting concrete aggregate with coal gangue. Despite the lower strength compared to regular gravel aggregate, coal gangue aggregate has the ability to absorb water during concrete mixing. This leads to a localized decrease in the water–cement ratio on the surface of coal gangue particles, ultimately enhancing the compactness of cement stone near the gangue aggregate surface. At the same time, because the surface of gangue particles is rough and has micropores, the bonding force between the gangue and cement stone is improved, and a hard cement shell is formed around the gangue. Restricting the transverse deformation of aggregate enhances the ultimate strength of gangue, resulting in coal gangue concrete having a strength comparable to ordinary concrete. These characteristics lay an important foundation for the resource utilization of coal gangue. Therefore, the production of cement concrete with coal gangue can not only improve the ecological environment of a mining area and reduce the occupation of the land but also save and protect the natural stone resources of our country and realize the properties of lightweight, high strength, and thermal insulation of concrete. It greatly improves the comprehensive utilization rate of coal gangue, thus promoting the two–way, harmonious, and sustainable development of the coal industry and construction industry. It can bring enormous environmental, economic, and social benefits [18,19,20]. According to the test findings [21], coal gangue concrete exhibits greater brittleness compared to ordinary concrete, and its resistance to deformation is inferior to that of ordinary concrete.

Adding fiber is an internationally recognized method that can effectively reduce the brittleness of concrete materials and improve their toughness. Combining various types and sizes of fibers can effectively inhibit the formation and spread of cracks in concrete at different structural levels, significantly enhancing the durability and fracture resistance of the material. The main component of a disposable mask is polypropylene, which has a low density, low melting point, and medium stiffness and strength. It is one of the most widely used resin materials [22], and it is also partly used in building materials. Xiao Qingyi et al. [23] found that, in the later phase of curing, the splitting strength, compressive resilience modulus, compressive strength, and water stability were enhanced by using a combination of polypropylene fiber and lime–fly ash–stabilized red clay. According to Zhang Zhitao [24], the addition of polypropylene fiber to gravel clay enhances the tensile strength and ultimate tensile strain of soil samples. The correlation improvement was directly proportional to the amount of fiber added. However, as the gravel content in gravel clay increases, the impact of fiber on the tensile strength noticeably diminishes. Huang Hua et al. [25] found the bridging effect of polypropylene fiber through electron microscope scanning, and an appropriate addition could effectively prevent the emergence and development of early microcracks in geopolymer concrete. However, the excessive addition of fiber would lead to the phenomenon of “reunion”, resulting in more holes in the concrete structure, and the improvement effect would be reduced or even detrimental. Mohammad Saberian et al. [26] used mask fiber–reinforced recycled concrete blocks and found that mixing a 1~2% mask fiber segment in recycled concrete aggregate could improve the strength and stiffness of reinforced soil. It was considered that the fiber is closely combined with soil particles, and the bridging effect of fiber hinders the further development of tension cracks. However, after adding more than 2% fiber, the excess fiber segment produced numerous voids, decreasing the strength and stiffness of the soil sample.

Latizia [27] and others used recycled polymer waste to produce fibers and replace aggregates in mortar to improve the extensibility of mortar, but a large amount of waste fibers also affected the hydration and increased the porosity of mortar, decreasing its compressive strength. S. Agyeman [28] cut recycled polypropylene plastic bags into concrete and compared them with finished fiber. This research showed that the fiber produced from recycled products could somewhat improve the performance of mortar, and it was more environmentally friendly. Yang Chengzhi [29] processed cement–woven bags composed of polypropylene into bundle fibers, mixed them with basalt fibers, and analyzed their effect on the mechanical properties of recycled concrete at different proportions. The inclusion of hybrid fibers could enhance the ratio of elastic strength and tension compression in recycled concrete, as well as improve its plasticity. Zhou Jinghai [30] treated waste polypropylene carpets with bundle fiber in concrete, which improved the pore structure of the cement paste, slowed water loss, and effectively reduced shrinkage deformation to prevent the occurrence of concrete cracks.

In Idrees M [31], mask waste was treated via fibrosis and square crushing. When 1% of mask fiber was added, the concrete showed better compressive and tensile strength, but when the fiber content of the mask waste exceeded 1.5%, the strength of the concrete decreased. Kilmartin Lynch S [32] performed a detailed study on the content of mask waste and found that the increase in compressive strength after adding mask waste fiber was due to the limiting effect of polypropylene fiber in the mask waste, which reduced the occurrence of cracks. When 0.25% mask waste fiber was added, the concrete strength decreased, which may have been due to the gap when 0.25% of the volume was replaced. Koniorczyk M [33] processed mask waste into a fiber shape under high temperature and high pressure, cut it into 5 mm fibers and added it to a concrete mixture. After undergoing 100 cycles of freezing and thawing, the concrete exhibited a marginal improvement in its compressive strength compared to the reference concrete, with a 5% increase observed in its 28–day compressive strength. Ali M [34] found that the fiber content of mask waste should not exceed 1% because a lower fiber content means that the fibers do not tangle or gather, and there is no homogeneity problem. As a result, the concrete exhibits increased tensile strength and enhanced resistance to cracking, thereby minimizing the infiltration of detrimental substances and enhancing its longevity. Alrshoudi F’s [35] study found that the drying shrinkage of a concrete specimen mixed with mask fiber waste is 29.5% lower than that of an ordinary mixture, which effectively prevents the cracks caused by shrinkage, and the bridging effect of waste polypropylene fiber has a high potential to resist impact load, thus reducing the brittleness of concrete and preventing the sudden failure of prefilled aggregate concrete specimens.

As mentioned above, mask waste fibers have higher tensile strength and greater flexibility and can be used as feasible alternative base and subbase materials. However, in existing research, polypropylene is widely used in concrete, and good results have been achieved, but research on using mask waste to enhance the performance of coal gangue concrete is rare. To realize the reuse of resources, this paper intends to apply recycled mask waste as adulterated fiber in coal gangue concrete. This paper explores the viability and factors that influence coal gangue concrete mixed with masks by conducting compression tests using various doping methods and mask contents. Studying the impact of masks on coal gangue concrete serves multiple purposes, including resource recycling, enhancing the concrete’s performance, and offering valuable insights into mitigating environmental pollution.

## 2. Test Instruments and Materials

### 2.1. Test Materials

Disposable medical mask waste and coal gangue were selected for the experiment. The mask consisted of two layers of nonwoven cloth and a layer of melt–blown cloth, all composed of polypropylene (PP). PP is non–toxic, odorless, resistant to acid, alkali, salt solution, and various organic solvents, and has a certain level of durability. The temperature range is –30 °C to 140 °C [36].

**Figure 1:**
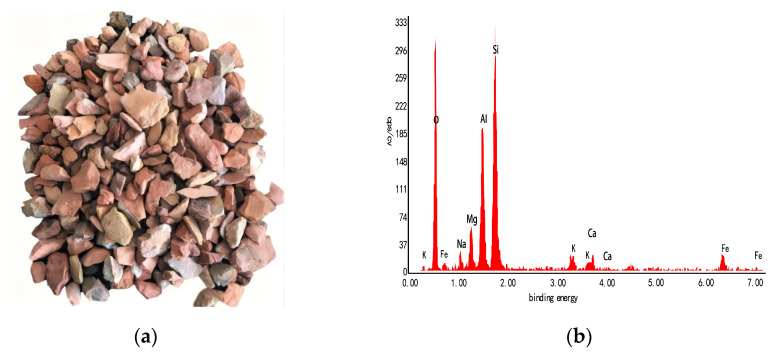
Coarse aggregate of coal gangue and its energy spectrum. (**a**) Spontaneous combustion coal gangue from Haizhou Ping’an Mine in Fuxin; (**b**) energy spectrum of coal gangue.

The coal gangue utilized in the experiment originated from the self–igniting coal gangue found at the Haizhou Ping’an Mine in Fuxin. Following the cleaning process, it exhibits a ceramic red or brick red color and possesses a porous structure. Figure 1 below displays the chemical composition, mineral composition, and micromorphology.

The X-ray fluorescence spectrometry analysis indicated that the primary chemical makeup of coal gangue coarse aggregate in the Fuxin coal mine consists of diverse inorganic substances and complexes. The inorganic substances mainly included minerals and water. Among the minerals composed of more than ten elements, SiO_2_ accounted for the largest proportion, with a content of 62.23%, followed by Al_2_O_3_, with a content of 17.51%. Other inorganic substances with unequal contents, such as Fe_2_O_3_, CaO, MgO, TiO_2_, K_2_O, and Na_2_O, were mainly composed of inorganic substances with unequal content, as shown in Table 1 below.

### 2.2. Test Instrument

**Figure 2:**
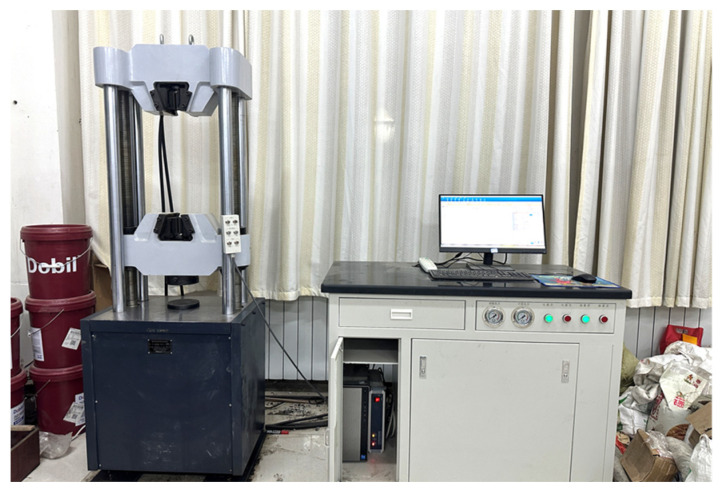
Test system.

Figure 2 displays the utilization of the WAW–300B microcomputer controlled electro–hydraulic servo universal testing machine manufactured by Shanghai Precision Instruments Co., Ltd., Shanghai, China during the experiment. The equipment is mainly composed of three parts: a mainframe, a servo–hydraulic press, and an electronic control system. It can be loaded under steady pressure, constant stress, and constant strain, and the maximum axial load is 700 kN.

## 3. Test Scheme

**Figure 3:**
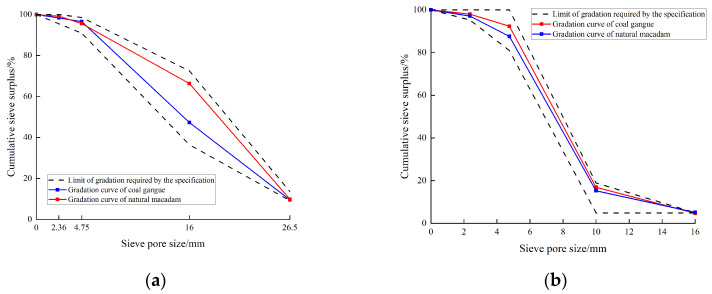
Coarse bone gradation curve. (**a**) 5~20 mm; (**b**) 5~10 mm.

After sampling, the coarse aggregate of coal gangue was manually crushed and then crushed twice by a jaw crusher. According to “Pebble and Macadam for Construction” (GB/T14685–2011) [37], coal gangue aggregate with particle sizes of 5~20 mm was selected to replace the natural macadam. The particle size of the coarse aggregate of the cylindrical specimen was 5~10 mm, and the particle gradation curve of the coarse aggregate is shown in Figure 3.

The raw materials needed for the test were prepared in accordance with the standards for sand and stone quality and test methods for ordinary concrete (JGJ52–2006) [38] and the code for the mix proportion design of ordinary concrete (JGJ55–2011) [39]:

(1) Cement: Fuxin Yingshan brand of ordinary Portland cement. The strengths are C20 and C30, and the relevant parameters are shown in Table 2;
(2) The fine aggregate was river sand, the maximum particle size was less than 4.75 mm, and the fineness modulus was 1.89. The related parameters are shown in Table 3;
(3) Coarse aggregate: coal gangue that is mechanically broken and meets the relevant standards;
(4) Superplasticizer: model CQJ–JSS powder polycarboxylic acid superplasticizer;
(5) Water: ordinary tap water;
(6) Table 4 displays the chemical composition and physical properties of the mineral admixtures, namely silica fume, fly ash, and slag powder;
(7) The disposable masks were sterilized and cut into strips after recycling, the bundle–like fiber was created by cutting off the nasal bridge and ear cord. And the performance parameters of the discarded masks is shown in Table 5.

**Figure 4:**
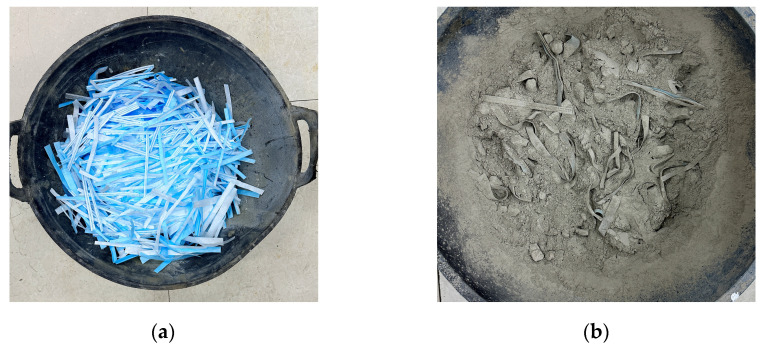
Concrete production process. (**a**) Mask fiber; (**b**) coal gangue concrete dry mix.

The experiment considered the mechanical characteristics of coal gangue concrete with varying mask content to investigate the relationship between strain, strength, and mask fiber doping. In the reference group, the coal gangue concrete exhibited strength grades of C20 and C30. To compare the effect of different content of hybrid masks on the mechanical behavior of concrete with reference to the existing research results on the adulteration of mask waste in concrete, five kinds of mixing modes of mask waste were designed on the basis of each reference group of coal gangue concrete, which were 0.5%, 1.0%, 1.5%, 2.0%, and 2.5% of the mass of concrete, abbreviated as DMGC–20–0.5, DMGC–20–1.0, DMGC–20–1.5, DMGC–20–2.0, DMGC–20–2.5 and DMGC–30–0.5, DMGC–30–1.0, DMGC–30–1.5, DMGC–30–2.0, and DMGC–30–2.5, respectively. For example, DMGC–20–0.5 represented mask–reinforced gangue concrete with a matrix strength of C20 and a hybrid mask content of 0.5% of the mass of the concrete, and OCGC represented ordinary gangue concrete. Figure 4a displays the cut bundle–like fiber after disinfecting, drying, and removing the nose bridge and ear cord from the discarded masks. Additionally, the mask fiber coal gangue concrete dry mix is shown in Figure 4b.

To improve the dispersion uniformity of the fiber, the mixing time of the DMGC mixture must be prolonged. During the blending procedure, the coarse and fine aggregates were mixed and stirred for 30 s. Subsequently, the cement and mineral admixtures of C20 (or C30) were added to the mixer, and the mixture was stirred continuously for 2 min. After mixing the mask fiber, the mixture was stirred for 2 min. Finally, the premixed water and superplasticizer were added to the mixer, and the mixture was stirred for 2 min. After stirring evenly, the DMGC mixture was promptly poured into a prism mold measuring 100 mm × 100 mm × 300 mm and compressed using a shaking table. Following a 24-h period at room temperature, the sample was taken out and transferred to a standard curing room with a temperature range of 20 ± 2 °C and a relative humidity exceeding 95% for a duration of 28 days. Table 6 displays the adulteration plan involving mask waste fiber in coal gangue concrete.

## 4. Test Results and Analysis

### 4.1. Mechanical Properties

**Figure 5:**
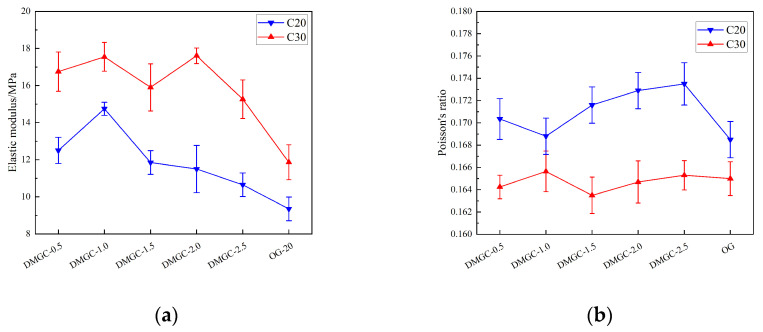
The variation of the Poisson ratio and elastic modulus of DMGC. (**a**) Elastic modulus; (**b**) Poisson ratio.

Figure 5 illustrates the impact of the quantity of mask fiber added on the mechanical characteristics of coal gangue concrete. Examining the C20 coal gangue concrete, it is evident that the addition of mask fiber enhances the compressive strength of the specimen. Specifically, the compressive strength increases by 2.3%, 4.1%, 5.6%, 1.2%, and −1.8% when 0.5%, 1.0%, 1.5%, 2.0%, and 2.5% mask fiber are added, respectively, in comparison to the reference without mask fiber. After adding 0.5%, 1.0%, 1.5%, 2.0%, and 2.5% mask fiber, the compressive strength of the specimen increases by 1.4%, 3.1%, 4.7%, 0.8%, and −1.3% respectively when compared to the unadded mask fiber reference with the C30 concrete strength. In conclusion, the inclusion of mask fiber does enhance the strength of coal gangue concrete, and a fiber dosage of 1.5% could yield the maximum strength for the concrete composed of coal gangue. The compressive strength of coal gangue concrete may decrease as the amount of fiber increases beyond a certain threshold, potentially due to the soft and flexible nature of the added fiber. During the blending procedure of coal gangue concrete, the fiber used as reinforcement has a tendency to flex and readily aggregate, resulting in an unequal dispersion of fibers within the coal gangue concrete, consequently causing voids to form. Consequently, the overall density of concrete composed of coal gangue is diminished, resulting in a decline in the concrete strength. When the fiber content is below 1.5%, it becomes easier to uniformly blend the reduced fiber content into the concrete. Additionally, the suppleness characteristic of the fiber itself strengthens the coal gangue concrete’s ability to resist cracks, primarily due to the fiber acting as a barrier against certain microcracks. Consequently, this enhances the impermeability and longevity of the coal gangue concrete. Furthermore, in cases where the concrete has a high level of strength, the impact of the fiber amount on its strength is comparatively less significant compared to low–strength concrete. This could be attributed to the fact that the strength of the mask is considerably inferior to that of the concrete. Hence, the growth becomes less apparent when the concrete’s strength is increased.

### 4.2. Stress–Strain Curve

**Figure 6:**
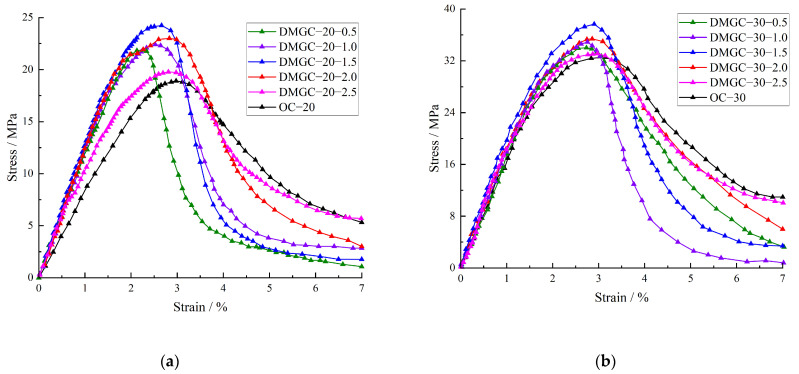
Stress–strain curves of DMGC. (**a**) C20; (**b**) C30.

The stress–strain curve of the DMGC is shown in Figure 6. Figure 6 shows that when the matrix strength is the same, the overall shape of the stress–strain curve of concrete obviously changes with the addition of mask fiber. For DMGC with different matrix strengths, with increasing matrix strength, the influence of mask fibers on the elastic deformation stage of the peak stress front line of the stress–strain curve decreases. For the coal gangue concrete reference group, with increasing matrix strength, the stress reduction rate and brittle failure characteristics increase after the peak stress of the stress–strain curve.

However, with the addition of mask fiber, the effect of matrix strength on the brittle failure characteristics of coal gangue concrete obviously decreases, further showing that adding mask fiber helps to improve the ductile deformation of coal gangue concrete. The strength of coal gangue concrete gradually increases as the fiber mass content rises from 0.5% to 1.5%. However, it decreases when the fiber content reaches 2.0% and 2.5%. At hybrid fiber volume contents of 2.0% and 2.5%, the slope of the elastic deformation phase in the stress–strain curve’s peak stress region decreases, leading to a significant increase in the ductile deformation of coal gangue concrete. As previously stated, this outcome should be achieved due to the excessive quantity of mask fiber, which reduces the even distribution of fiber within the coal gangue concrete matrix, leading to accumulation and clustering. Additionally, the mixing process becomes susceptible to the introduction of air bubbles, resulting in an increase in the internal flaws of the coal gangue concrete and a decrease in its resistance to deformation.

Figure 6 demonstrates that the elastic modulus of DMGC changes depending on the mass content, type, and strength of the cover fiber matrix. Since the elastic modulus indicates the material’s resistance to deformation in its undamaged state, the internal flaws of coal gangue concrete have not yet formed, and the reinforcing effect of fibers has not been demonstrated. Hence, the impact of mask fiber on the flexural modulus of coal gangue concrete differs from its impact on compressive strength. Compared to the coal gangue concrete reference group, the elastic modulus of DMGC–20–0.5 and DMGC–30–0.5 increases by 5.52% and 6.93%, respectively, when the fiber content in the mask is 0.5%. Additionally, the compressive strength of DMGC–20–0.5 and DMGC–30–0.5 increases by 2.5% and 1.4%, respectively. Other cases are shown in Figure 6. By observing the curing process of coal gangue concrete, it becomes evident that the inclusion of mask fiber effectively restrains the shrinkage and cracking of the concrete during the hardening phase. Hence, the inclusion of mask fiber aids in diminishing the initial flaws in coal gangue concrete; however, surpassing a specific ratio will diminish the elastic modulus and compressive strength of the concrete made from coal gangue. The observed outcome is due to the excessive incorporation of mask fiber, which leads to an increase in internal flaws and a decrease in the elastic modulus of coal gangue concrete. Furthermore, this outcome elucidates the reason behind the reduction in elastic modulus and compressive strength of coal gangue concrete when the quantity of mask fiber exceeds 1.5%.

### 4.3. Law of Energy Evolution

**Figure 7:**
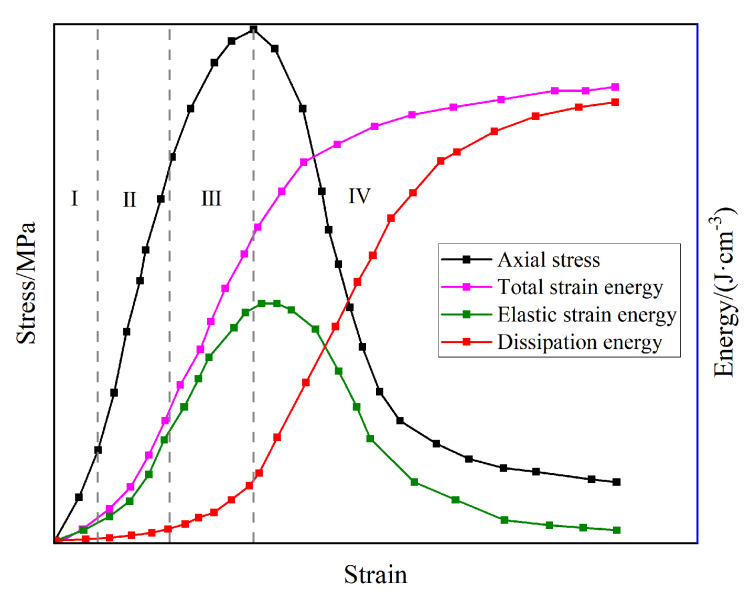
Energy evolution characteristics of mask–reinforced gangue concrete (DMGC).

The stress–strain relationship and energy evolution characteristics of coal gangue concrete are shown in Figure 7. As shown in Figure 7, the energy evolution characteristic curve of coal gangue concrete can be divided into four stages. (I) The primary crack closure stage: The primary crack in coal gangue concrete is closed under the initial load, and the total strain energy is small. No new cracks are formed in the coal gangue concrete, and the dissipation energy is small. (II) The linear elastic stage: In this stage, coal gangue concrete is in the stage of elastic deformation, and no new cracks are formed in coal gangue concrete, so the dissipation energy is still small. The coal gangue concrete stores the total strain energy as elastic strain energy. (III) The stage of rapid crack expansion: In this stage, new cracks gradually appear in the coal gangue concrete, part of the total strain energy is converted into dissipative energy, and the increasing rate of elastic strain energy decreases. (IV) Crack penetration stage: After the peak stress, the cracks in the coal gangue concrete gradually run through, forming macroscopic cracks and material failure. At this time, due to the gradual decrease in the load, the increasing rate of the total strain energy decreases. The elastic strain energy stored in coal gangue concrete dissipates as dissipated energy due to the penetration of cracks. Therefore, the elastic strain energy decreases gradually, and the dissipated energy increases rapidly.

**Figure 8:**
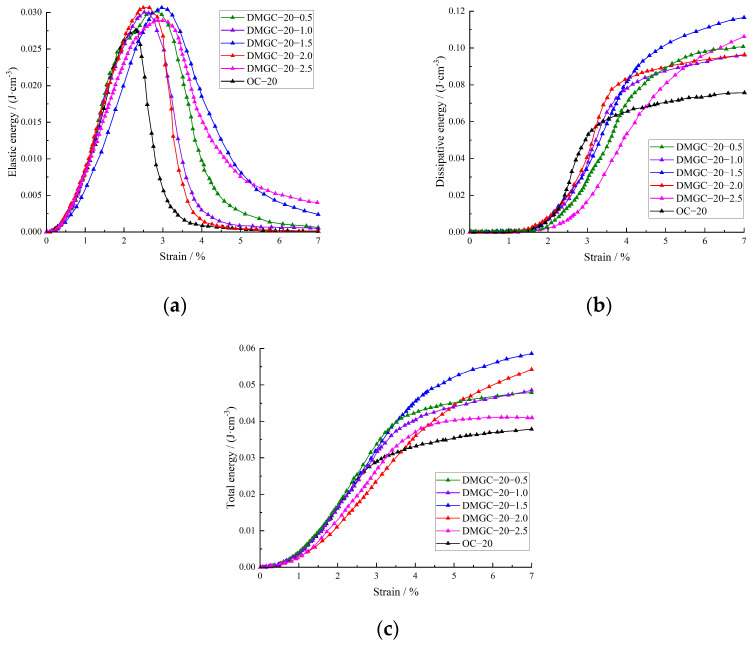
Energy evolution law of DMGC with a matrix strength grade of C20. (**a**) Elastic strain energy; (**b**) dissipated energy; (**c**) total strain energy.

**Figure 9:**
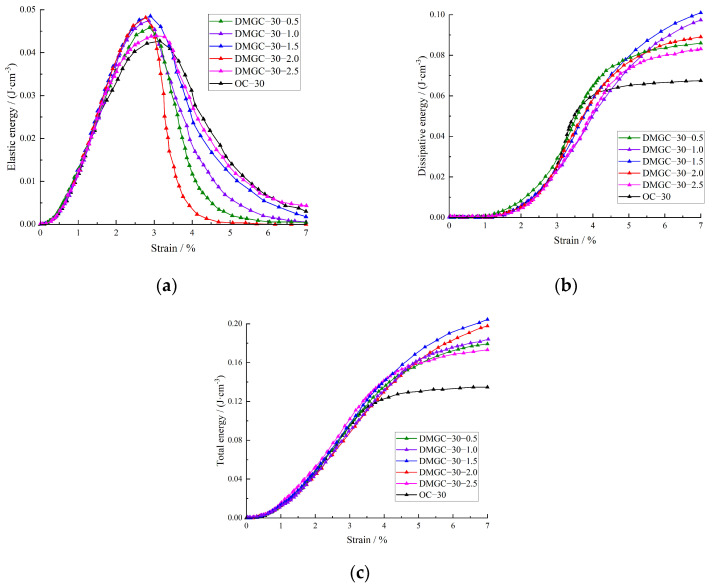
Energy evolution law of DMGC with a matrix strength grade of C30. (**a**) Elastic strain energy; (**b**) dissipated energy; (**c**) total strain energy.

Figure 8 and Figure 9 represent the strain energy evolution of DMGC with different matrix strength grades; the total strain energy of the DMGC is comparable to the dissipated energy when the matrix strength is identical. The impact of the fiber mass content on the overall strain energy and dissipated energy of coal gangue concrete is minimal when the mass content of mask fiber is 0.5%. Nevertheless, as deformation increases, the growth rates of both the overall strain energy and the dissipated energy in coal gangue concrete are amplified by the doping amount of mask fiber.

When the final deformation stage is reached, the total strain energy and the dissipated energy for DMGC with different matrix strength grades and different fiber mass contents compared with benchmark gangue concrete are as follows: for DMGC–20–0.5 and DMGC–30–0.5, the total strain energy is increased by 12.02% and 15.12% respectively, and the dissipated energy is increased by 11.97% and 14.83% respectively; for DMGC–20–1.0 and DMGC–30–1.0, the total strain energy is increased by 18.15% and 19.92% respectively, and the dissipated energy is increased by 17.94% and 19.22% respectively; for DMGC–20–1.5 and DMGC–30–1.5, the total strain energy is increased by 25.78% and 27.56% respectively, and the dissipated energy is increased by 25.34% and 26.22% respectively; for DMGC–20–2.0 and DMGC–30–2.0, the total strain energy is increased by 16.65% and 18.23% respectively, and the dissipated energy is increased by 16.11% and 17.12% respectively; for DMGC–20–2.5 and DMGC–30–2.5, the total strain energy is increased by 5.69% and 7.23% respectively, and the dissipated energy is increased by 4.21% and 6.42% respectively.

The above analyses show that when the matrix strength grade is C20 with an increase in the mass content of mask fiber, the total strain energy will first increase and then decrease; the peak increase appears when the mass content of mask fiber is 1.5%; the variations of dissipated energy with the increase in fiber content show a similar trend to the total strain energy, and what is more, the peak increase also appears at a fiber content level of 1.5%. The above trends also apply when the matrix strength grade is C30.

The elastic strain energy variation curve indicates that prior to reaching the maximum elastic strain energy, the impact of mask fiber content on the elastic strain energy of coal gangue concrete is comparable to that of total strain energy and dissipated energy. Following the maximum elastic strain energy, the elastic strain energy of coal gangue concrete in the control group declines rapidly, suggesting a rapid crack propagation rate and a prominent brittle failure characteristic. By incorporating fiber, the gradual reduction in elastic strain energy decreases, leading to an increase in ductile failure characteristics, which is further amplified with higher fiber content. The crack–bridging impact of mask fiber partially inhibits the speed of crack propagation and penetration while enhancing the ductile failure properties of coal gangue concrete. Comparing Figure 8 and Figure 9 reveals that as the matrix strength increases, the overall strain energy, maximum elastic strain energy, and dissipated energy progressively rise. Additionally, the influence of mask fiber content on the energy evolution traits of coal gangue concrete diminishes over time. The findings indicate that enhancing the matrix’s strength reduces the impact of mask fiber on the efficacy of coal gangue concrete. DMGC has an elastic strain energy conversion of α = Ue/U and a dissipated energy conversion of β = Ud/U.

**Figure 10:**
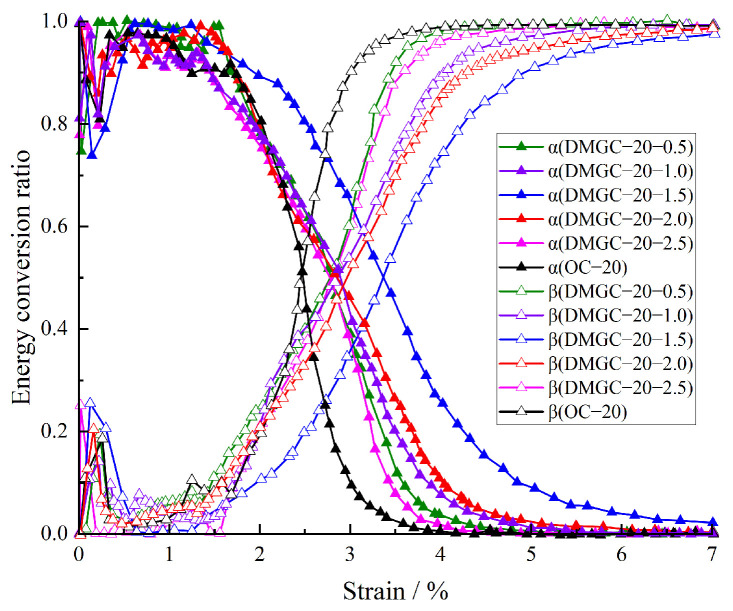
Energy conversion ratio of DMGC.

The energy conversion rate of DMGC is shown in Figure 10. Figure 10 shows that when the strength of the matrix is the same, the elastic strain energy conversion of DMGC decreases, and the dissipated energy conversion increases because the closed friction of the primary crack consumes a certain amount of energy. In the stage of elastic deformation, the internal interface of coal gangue concrete is increased due to the addition of mask fiber. In the process of loading, the dislocation and friction of the interface consume a certain amount of energy. Therefore, adding mask fiber reduces the elastic strain energy conversion rate of coal gangue concrete and increases the dissipated energy conversion rate. In the stage of rapid crack expansion, the crack–bridging effect of fiber and the addition of fiber reduces the decreasing rate of the elastic strain energy conversion rate and the increasing rate of the dissipated energy conversion rate of coal gangue concrete. When the crack penetration stage is reached, the crack tends to run through, and the crack limiting effect of fiber is brought into play to a greater extent, which reduces the crack penetration rate of coal gangue concrete, substantially reduces the decreasing rate of the elastic strain energy conversion rate of coal gangue concrete and the increasing rate of the dissipated energy conversion rate, and increases the ductile deformation of coal gangue concrete. With increasing matrix strength, the concentration of the elastic strain energy conversion rate and dissipated energy conversion curve of the DMGC increases, further indicating that the increase in matrix strength weakens the effect of mask fiber on the properties of coal gangue concrete.

### 4.4. Establishment of the Constitutive Model

The nonlinearity of concrete materials is caused by two main energy dissipation mechanisms: the evolution of microcracks and plastic yield flow. Plastic flow produces irrecoverable plastic deformation, and the propagation of microcracks decreases the stiffness and strain softening of concrete [40,41,42,43]. Domestic and foreign scholars [44,45,46,47,48,49] have used continuum mechanics to add damage variables to the constitutive model to reflect the irreversible damage evolution process of materials.

According to Lemaitre’s strain equivalence principle, the following constitutive equations of rock damage are established:

(1) $$\sigma_{*}=E\varepsilon (1-D)$$

Since quasibrittle materials like concrete and rock follow the Weibull distribution in terms of their meso–heterogeneous mechanical properties and damage, it is possible to further represent the damage variable in the following equation:

(2) $$D=1-\exp\left[-{\left(\frac{F}{F_{0}}\right)}^{m}\right]$$

Therefore:

(3) $$\sigma =E\varepsilon \exp\left[-{\left(\frac{F}{F_{0}}\right)}^{m}\right]$$

In this formula, *σ* is the effective stress matrix, *E* is the elastic modulus matrix, ε is the elastic strain matrix, D is the damage factor of rock, *F* is the distribution variable of the microelement failure Weibull distribution, and *F*_0_ and *m* are the scale parameters and shape parameters, respectively.

For any stress principal stress, $\sigma_{i}\;\left(i=1,\;2,\;3\right)$ is the effective shear stress on the octahedron, which can be expressed as:

(4) $$\tau_{oct}=\frac{1}{3}\sqrt{{\left(\sigma_{1}-\sigma_{2}\right)}^{2}+{\left(\sigma_{1}-\sigma_{3}\right)}^{2}+{\left(\sigma_{2}-\sigma_{3}\right)}^{2}}$$

The effective stress after damage is taken instead of the above principal stress, and the shear stress on the octahedron is taken as the microelement strength index:

(5) $$F=\frac{1}{3}\sqrt{{\left(\sigma_{*1}-\sigma_{*2}\right)}^{2}+{\left(\sigma_{*1}-\sigma_{*3}\right)}^{2}+{\left(\sigma_{*2}-\sigma_{*3}\right)}^{2}}$$

Under the action of uniaxial force, $\sigma_{2}=\sigma_{3}=0$, the combination of Equation (5) and Equation (3) can be obtained:

(6) $$\sigma =E\varepsilon \exp\left[-{\left(\frac{\sqrt{2}\left(1-\mu \right)E\varepsilon }{3F_{0}}\right)}^{m}\right]$$

The stress–strain relationship of mask fiber–doped concrete can be solved according to Equation (6). To facilitate the fitting solution, appropriate changes must be accommodated for in Equation (6). As shown below, Equation (6) is first transformed into the following form:

(7) $$\frac{\sigma }{E\varepsilon }=\exp\left[-{\left(\frac{\sqrt{2}\left(1-\mu \right)E\varepsilon }{3F_{0}}\right)}^{m}\right]$$

Then, the logarithm on both sides can be obtained:

(8) $$\ln\frac{\sigma }{E\varepsilon }=-{\left(\frac{\sqrt{2}\left(1-\mu \right)E\varepsilon }{3F_{0}}\right)}^{m}$$

The following equations are derived via a transformation:

(9) $$F_{0}{\left(-\ln\frac{\sigma }{E\varepsilon }\right)}^{\frac{1}{m}}=\frac{\sqrt{2}\left(1-\mu \right)E\varepsilon }{3}$$

Then, the above equation can eventually be changed into the following simplified equation:

(10) $$F_{0}\Psi^{\alpha }=\Omega$$

In this formula,

$$\Psi =-\ln\frac{\sigma }{E\varepsilon },\;n=\frac{1}{m},\;\Omega =\frac{\sqrt{2}\left(1-\mu \right)E\varepsilon }{3},$$

After fitting the test curve according to the above equation, the results obtained are shown in Table 7 and Table 8 below.

**Figure 11:**
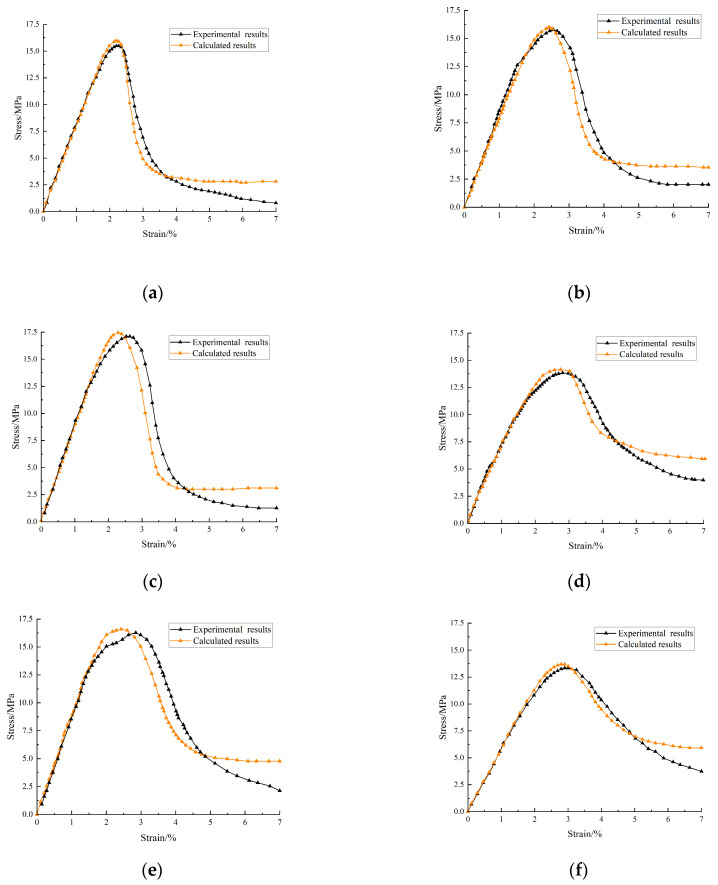
Energy evolution law of DMGC with a matrix strength grade of C20. (**a**) DMGC–20–0.5; (**b**) DMGC–20–1.0; (**c**) DMGC–20–1.5; (**d**) DMGC–20–2.0; (**e**) DMGC–20–2.5; (**f**) OG–20.

**Figure 12:**
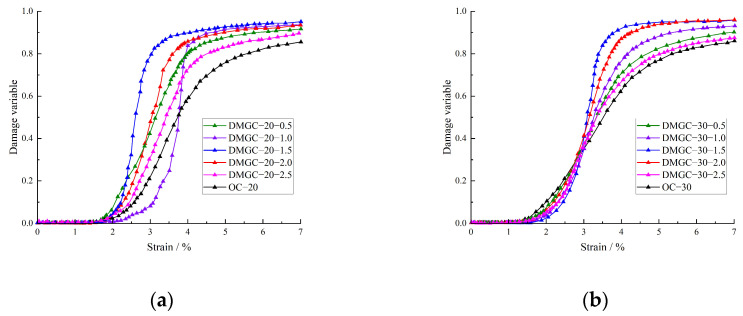
Damage evolution characteristics of DMGC. (**a**) C20; (**b**) C30.

Figure 11 shows that the selected damage constitutive model fits well with the test results. The model curve closely aligns with the test curve prior to reaching the maximum stress point but starts to diverge afterward. The constitutive model curve selected before the inflection point of the decreasing section of the fiber–doped coal gangue concrete stress–strain curve and the test curve also has a high degree of fit. After the inflection point of the descending section, the stress magnitude of the model curve swiftly diminishes, leading to the occurrence of the stress reduction phenomenon. When the strain increases to a certain extent, the stress tends to zero, but the decline rate of the test curve is significantly slower than that of the model curve. The main reason for this phenomenon is that, in the descending section of the curve, the damage value of gangue concrete exceeds a certain critical limit. The damage grows rapidly, and the mechanical properties of the material deteriorate rapidly. A large amount of new damage further occurs, causing the bearing capacity to rapidly decrease. The damage evolution characteristics of DMGC are illustrated in Figure 12 below.

## 5. Conclusions

The mechanical properties of coal gangue concrete can be improved by using a suitable amount of masks. When the mass fraction of the disposable mask accounts for 1.5% or less of the total mass of coal gangue concrete, increasing the amount of mask added helps to improve the compressive strength, elastic modulus, and deformation resistance of coal gangue concrete. It promotes improvement in the mechanical properties of concrete. The anti–microcracking property of mask fiber helps to improve the impermeability and oxidation resistance of coal gangue concrete, allowing for an increase in the service life of coal gangue concrete in wet or immersed environments. From an energy perspective, the mask fiber reduces the elastic strain energy release rate of coal gangue concrete and increases the dissipated energy of coal gangue concrete, increasing the total strain energy of coal gangue concrete. Masks improve the ductile deformation and toughness of coal gangue concrete.

## Figures and Tables

**Table 1:** Chemical composition and content of coal gangue in the Fuxin area.

Composition	SiO_2_ /%	Al_2_O_3_ /%	Fe_2_O_3_ /%	CaO /%	MgO /%	TiO_2_ /%	K_2_O /%	Na_2_O /%
Coal gangue	62.23	17.51	3.26	1.82	1.91	0.81	3.33	2.37

**Table 2:** Cement parameters in the Fuxin area.

	Performance Index					
	Stability	Specific Surface Area /(m^2^/kg)	Initial Setting Time /h	Final Setting Time /h	28 d Compressive Strength/MPa	28 d Flexural Strength/MPa
PO42.5	Qualified	344	3.24	4.5	46.7	7.1

**Table 3:** Fine aggregates in the Fuxin area.

Detection Index	Apparent Density /(kg/m^3^)	Packing Density /(kg/m^3^)	Porosity Ratio /%	Water Content /%	Fineness Modulus
**Measured Value**	61.25	17.68	3.25	1.74	1.89

**Table 4:** Chemical composition and physical properties of mineral admixtures.

Material	Mass Fraction/%									Density /(g·cm^−3^)	28 d Active Index/%	Specific Surface Area/(m^2^·kg^−1^)
	CaO	SiO_2_	A1_2_O_3_	Fe_2_O_3_	MgO	SO_3_	Other	Water	Ignition Loss			
Silica fume	1.63	85.04	0.97	1.04	0.32	/	10.00	0.06	5.48	2.10	131	23,000
Fly ash	21.14	35.71	16.57	8.92	1.41	1.94	12.49	0.20	2.85	2.35	97	340
Slag	34.11	34.65	14.21	0.49	11.15	1.00	3.74	0.70	0.30	2.86	104	410

**Table 5:** Performance parameters of the discarded masks.

Proportion	Melting Point/°C	Water Adsorption /%	Tensile Strength /Mpa	Fracture Tensile Strength /Mpa	Elongation at Break /%	Fracture Force /N
0.93	169	9.5	4.26	4.18	20.6	19.46

**Table 6:** Adulteration scheme of mask waste fiber in coal gangue concrete.

Serial Number	Fiber Length/mm	Fiber Content/%
Blank control group (OG)	0	0
DMGC–0.5	10	0.5
DMGC–1.0	10	1.0
DMGC–1.5	10	1.5
DMGC–2.0	10	2.0
DMGC–2.5	10	2.5

**Table 7:** Parameter values of the damage constitutive model under uniaxial compression.

Serial Number	Peak Stress /MPa	Peak Strain /%	Experimental Modulus of Elasticity /MPa	Theoretical Modulus of Elasticity /GPa	Experimental Value/Theoretical Value	*F*_0_	*m*
OC–20	21.18	2.97	7473	10,098	0.74	0.003105	2.2623
DMGC–20–0.5	24.38	2.23	10,932	14,975	0.73	0.003422	2.2301
DMGC–20–1.0	25.10	2.52	11,456	14,687	0.78	0.003178	2.2430
DMGC–20–1.5	27.15	2.58	12,195	16,264	0.75	0.002934	1.8510
DMGC–20–2.0	25.76	2.73	11,181	14,711	0.76	0.003425	1.7426
DMGC–20–2.5	21.15	2.83	8972	11,215	0.80	0.003601	2.1022

**Table 8:** Damage constitutive equation and damage evolution equation under uniaxial compression.

Serial Number	Damage Constitutive Equation	Damage Evolution Equation
OC–20	$\sigma =7473\varepsilon \;exp\left[-{\left(\varepsilon /0.003105\right)}^{2.2623}\right]$	$D=1-exp\left[-{\left(\varepsilon /0.003105\right)}^{2.2623}\right]$
DMGC–20–0.5	$\sigma =10932\varepsilon \;exp\left[-{\left(\varepsilon /0.003422\right)}^{2.2301}\right]$	$D=1-exp\left[-{\left(\varepsilon /0.003417\right)}^{2.2311}\right]$
DMGC–20–1.0	$\sigma =11456\varepsilon \;exp\left[-{\left(\varepsilon /0.003178\right)}^{2.2430}\right]$	$D=1-exp\left[-{\left(\varepsilon /0.003178\right)}^{2.2430}\right]$
DMGC–20–1.5	$\sigma =12195\varepsilon \;exp\left[-{\left(\varepsilon /0.002934\right)}^{1.8510}\right]$	$D=1-exp\left[-{\left(\varepsilon /0.002934\right)}^{1.8510}\right]$
DMGC–20–2.0	$\sigma =11181\varepsilon \;exp\left[-{\left(\varepsilon /0.003425\right)}^{1.7426}\right]$	$D=1-exp\left[-{\left(\varepsilon /0.003425\right)}^{1.7426}\right]$
DMGC–20–2.5	$\sigma =8972\varepsilon \;exp\left[-{\left(\varepsilon /0.003601\right)}^{2.1022}\right]$	$D=1-exp\left[-{\left(\varepsilon /0.003601\right)}^{2.1022}\right]$

## Data Availability

Data are contained within the article.
